# Illustrations of the Contagious Nature of Puerperal Fever with Remarks on Its Treatment, and on Contagion and Infection

**Published:** 1848-08

**Authors:** S. Beecrofft

**Affiliations:** Hyde


*Illustrations of the Contagious Nature of Puerperal Fever with Remarks on its Treatment, and on Contagion and Injection. By* S. Beecrofft, *Esq., M.R.C.S.C.E., Hyde.*—I lay the following cases and remarks before the profession, from a desire to render my mite towards elucidating the true nature of contagious diseases. I think that of late there has been manifested a disposition to decry the influence of contagion; this probably arises from all epidemic diseases having been considered, by our earlier predecessors, to have been propagated thereby. I am afraid we are acting equally rashly with them, by rushing into the opposite extreme, and proportionally denying its agency. I do not apply such remarks to the class of cases I am about to relate, for I believe that most of us agree in the contagious character of puerperal fever, but I wish to show how careful we ought to be in refusing to acknowledge the influence of contagion. For some time it was denied to this class of diseases, and even now is by the French school. Not long since, there was the same disposition to deny that typhus was capable of being communicated by contagion, but I think, from the number of medical men who have lately fallen its victims, that question is finally set at rest.

I would state my opinion that all diseases originating from a pestiferous virus entering into the body, and poisoning the blood, should be set down as capable of transmitting the same virus to others, and thus communicating the disease. I cannot see why the animal body cannot expel from it that which enters it, and thus become the medium of infection. And here I would observe that the distinction made by some of our medical authors, between contagion and infection, is unnecessary, for we know that the skin (though in a minor degree than the lungs) has the property of absorption, and therefore we must come to the opinion that infection may enter the body by contact. There can be no doubt that depraved food, bad air, want of cleanliness, &c., are powerful means of propagating epidemic disease; but I question if it is generally generated by these agents. It may be owing to our ignorace, that we lay such stress on these perceptible causes, and trust not the unknown, merely from not being able to detect them in our present imperfect knowledge of chemistry. The preceding remarks arose from reading that the Sanitary Commission had come to the opinion that cholera is not contagious, which opinion appears to me to be rash and not trustworthy, until we better know what the true principle of contagion is. Having already encroached much on the valuable space of THE LANCET, I shall condense the following cases, and therefore shall not enter into the symptoms of each case :—

CASE 1.—Mrs. H.——, aged thirty-four years, of a gross, lax, habit, troubled with erysipelas nasi, was delivered, after an easy labour of her fifth child, on Saturday, Nov. 28, 1846, (the night of a violent snow-storm, and when extreme cold set in.) She was seized with vomiting and purging, and died on the morning of the eighth day after delivery. I attributed her death to exhaustion caused by the vomiting and purging, which I thought was brought on by carelessness in living—taking fermented liquor, and eating goose, beef, &c., on the second and third days after delivery.

I had permission to make a post-mortem examination, which was instituted fifty-five hours after death, but it was not so minute as it otherwise would have been, from my assistance being urgently requested at the case of convulsions which I will shortly describe. The body was in good condition; redness about the nose, of a slight dingy hue ; the right side was contracted ; the lungs and heart were healthy; blood very dark and fluid ; the stomach large, with considerable redness of its mucous coat, near the pylorus ; liver enlarged, very dark, easily stripped of its peritonæl coat, and breaking up like a soft coagulum ; gall bladder contained some dark fluid, similar to what had been ejected ; peritonæum perfectly healthy ; not the least effusion in the abdomen, nor any signs of inflammation of the peritonæum covering the viscera ; bowels healthy, in some parts congested ; kidneys healthy ; uterus contracted, containing nothing, but its internal surface coated with a dark viscid substance ; its walls were perfectly healthy, and I perceived no condition of any of the organs to account for death, unless it was the diseased state of the liver.

CASE 2.—Mrs. G——, aged twenty-six years, troubled with erysipelas nasi ; delivered of her third child, labour easy, twelve P.M., Dec.

6th. She slept well during the night; in the morning, about eight o'clock, complained of headach, and soon after vomited some bilious, dark, looking fluid. At ten o'clock, after vomiting, convulsions set in, and my attendance was required, whilst making the previous post-mortem examination. Although active treatment was resorted to, the convulsions continued, and she died at ten P.M. the same day, twenty-two hours after delivery,

CASE 3.—Mrs. B——, aged twenty years, healthy; delivered of her first child, at two A.M., Dec, 9th. Rigors came on during the following day, with well-marked symptoms of puerperal fever, and she died on the 15th, six days from delivery, pus being deposited in the shoulder and elbow the day prior to death.

CASE 4.—Mrs. N——, aged thirty-one years; not well for some weeks; suffering from cough and occasional abdominal pains, which resembled those of labour; but for some days before labour came on she went about her usual household duties. I went with the husband from the previous case, and she was delivered after an easy labour, of her fourth child, about three o'clock the same morning. Puerperal fever came on, and she died on the 11th of December, the day but one after delivery.

I now thought the cases to be contagious, and did not attend another labour until the 20th. After using every precaution, I on that day attended Mrs. E.——, who did well, and Mrs. H.——, the subject of the following case:—

CASE 5. Mrs. H——, aged thirty, suffering from varicose ulcer of the leg, surrounded with considerable redness, of an erysipelas nature, was delivered of her third child, after an easy labour on the morning of December 20th. On the fourth day after confinement, fever set in, and she died on the following Sunday, (Dec. 27th.)

CASE 6.—I was called to attend Mrs. M——, aged twenty-seven years, of her third child. The child was born, and the cord divided, when I arrived at the house; and on placing my hand on the abdomen, the uterus contracted, and the placenta was expelled. Her sister, who lived in the house, was suffering from typhus; but this patient ailed nothing prior to delivery. Puerperal fever set in the day after delivery, and she died the same as the preceding case.

I now gave up attending midwifery, went from home for a time, and again attended a labour on January 16th, 1847, from which time to Jan. 31st I attended seven cases. Two of them difficult, and required turning, and the other the use of the forceps, which I employed for a medical friend. All of these cases did well. The weather had been warmer; but at the latter part of the month it became very cold. On the morning of January 31st I attended

CASE 7.—Mrs. W——, aged twenty-five years; disfigured with erysipelas nasi, spreading to the cheeks; had seven rigors during labour, and chills the day previous; labour easy. Adynamic puerperal fever set in, and she died on the morning of February 5th, the face looking horrible from the extreme blackness of the erysipelatous parts.

I saw no more of the disease, though occasionally alarmed by rigors following delivery, excepting an anomalous case which occurred to a Mrs. F——, who was delivered on the first of April, she having just recovered from fever. The woman did well for the first fortnight, but was forced to exert herself in her household duties, being unable to pay for a nurse. She sent for me on April 23rd, and stated that she had been out for a distance of two miles, the week before, and had not been well since. The lochia had not ceased, and for some days she said that the discharge was very copious and disagreeable. She was then suffering from phlebitis, a phlegmon existing over the right elbow and hip, and she died on the 29th, having every symptom of puerperal fever.

In considering over the preceding cases, I think there cannot be any doubt that a contagious principle was generated by Mrs. H.——, (the first case,) which was conveyed to my third case, and thus the puerperal fever was originated; and I cannot help thinking the second case (convulsion) had some connection with that of Mrs. H——. The third, fourth, fifth, and sixth cases show the contagious nature of the disease, and the seventh and last cases, also, that puerperal fever may be propagated either by direct contagion, or by atmospheric influence acting on those who may be predisposed to the disease. The prevalence of erysipelas at the same time, and the tendency manifested to it in the cases related, strengthen the opinion that puerperal fever and erysipelas are the same disease, one being capable of producing the other. The symptoms presented by these puerperal cases evidenced that the disease was dependent upon a poisoned state of the blood, and not in any particular organ. There were a small, feeble, and very rapid pulse, anxiety and a sense of uneasiness about the heart, hurried breathing, cough; in some of the cases, purulent expectoration, and profuse sweating. Some were troubled with vomiting and purging; no tenderness of the abdomen, but on pressing the uterus, which was rather larger than usual, pain was produced. There was no swelling or tympanitis when death took place within the fourth day; but in those who lived longer, the abdomen began to enlarge a short time before death. The lochia was deranged, and occasionally scanty, and in one case retention of urine existed. The cases were distinctly of the adynamic kind, presenting signs of a violent shock to the whole system. With respect to the treatment of such a form, (not puerperal peritonitis,) I should be inclined to follow the advice of those who do not recommend bleeding. I tried it in two cases, but it did no good. I would assist nature by acting on the principle of elimination. A mild emetic might be serviceable at the commencement by unloading the biliary ducts, followed by frequent doses of calomel; for I believe the liver is the most important organ to call to our aid in expelling the poison, and it is probable, from its office as a depurative of the blood, that it presents a morbid appearance after death, and thus in various diseases, (cholera for instance,) this has been said to be the cause, when it ought only to rank as the effect of the disease. Diaphoretics also might assist; Dover's powder I found useful in calming the patient and relieving pain; indeed, every means of eliminating the poison from the blood might be employed, whilst at the same time the system must be supported by ammonia, wine, beef tea, &c.—*Lancet.*

